# Phytotoxic Effects of Commercial *Eucalyptus citriodora*, *Lavandula angustifolia*, and *Pinus sylvestris* Essential Oils on Weeds, Crops, and Invasive Species

**DOI:** 10.3390/molecules24152847

**Published:** 2019-08-05

**Authors:** María Dolores Ibáñez, María Amparo Blázquez

**Affiliations:** Departament de Farmacologia, Facultat de Farmàcia, Universitat de València. Avda. Vicent Andrés Estellés s/n, 46100 Burjassot, València, Spain

**Keywords:** *E. citriodora*, *L. angustifolia*, *P. sylvestris*, essential oils, GC–MS, phytotoxicity

## Abstract

Background: essential oils are well known for their pharmacological effectiveness as well as their repellent, insecticide, and herbicide activities. The emergence of resistant weeds, due to the overuse of synthetic herbicides, makes it necessary to find natural alternatives for weed control. The aim of this study was to evaluate the phytotoxic effects of *Eucalyptus citriodora*, *Lavandula* *angustifolia*, and *Pinus sylvestris,* three common commercial essential oils, on weeds (*Portulaca oleracea*, *Lolium multiflorum*, and *Echinochloa crus-galli*), food crops (tomato and cucumber), and the invasive species *Nicotiana glauca*. Methods: to determine herbicidal effects, essential oils were tested at different concentrations (0.125–1 µL/mL). The index of germination and seedling length data were recorded over 14 days. Results: the in vitro assays showed that *L. angustifolia* with linalool (38.7 ± 0.1%), 1,8-cineole (26.5 ± 0.1%), and camphor (14.2 ± 0.1%) as the main compounds showed the most phytotoxic effects affecting seed germination in weeds and tomato, and the aforementioned invasive species. *L. multiflorum* was the most sensitive weed, particularly to lavender essential oil, which decreased the growth of its hypocotyl and radicle by 87.8% and 76.7%, respectively, at a dose of 1 µL/mL. Cucumber was the most resistant food crop, with no significant reduction observed in seed germination and hypocotyl growth with *E. citriodora* and *L. angustifolia* essential oils. Conclusions: lavender essential oil represents a promising candidate for the development of effective and safe herbicides in the management of *L. multiflorum* affecting cucumber crops.

## 1. Introduction

The particular characteristics of essential oils—natural mixtures of volatile compounds—provide them with certain pharmacological properties, including their well-known antibacterial, antioxidant, anti-inflammatory, and cancer chemoprotective effects, as well as their repellent, herbicidal, and insecticidal biological activities [1,2,3,4], which have led to valuable applications in human health, food, and cosmetics industries, and in environment and agriculture. Certain essential oils have already demonstrated their influence on both the seed germination and seedling growth of weeds [5,6]. In this regard, origanum (*Origanum vulgare* L.) essential oil with carvacrol as its main compound has exhibited a significant inhibitory effect against seed germination and seedling growth of common purslane, Italian ryegrass, and barnyardgrass at a range of concentrations (0.125–1 µL/mL), as well as against *Sinapsis avensis* at 2 µL/mL and also Johnson grass (*Sorghum halepense* L.) [7,8,9]. *P. sylvestris* exhibited some inhibition of the early root growth of *Cassia occidentalis* (L.) Link. [10], and *E. citriodrora* essential oil affected the development of certain weeds, particularly the seed germination of *Amaranthus viridis* L. [11]. Among weeds, the following deserve special attention: (i) common purslane (*Portulaca oleracea* L.), an annual weed commonly affecting cultivated land, protected agriculture, forests, plantations, and orchards, where it competes for resources with many field crops, including cruciferous crops, potato, and tomato, among others [12]; (ii) Italian ryegrass (*Lolium multiflorum* Lam.), an annual to biennial poaceous species largely spread globally due to its cultivation as a pasture grass [13], which has developed considerable resistance against glyphosate and other synthetic herbicides as an acetolactate synthase (ALS) inhibitor [14,15]; and (iii) barnyardgrass (*Echinochloa crus-galli* (L.) Beauv.), considered one of the world’s worst weeds, affecting agricultural land and grasslands as well as irrigation channels and wetlands, being, in fact, a very serious weed in rice crops [16]. In addition, other species, such as *Nicotiana glauca* Graham, native to South America and naturalized in several countries, have a high invasion potential to disturb ecosystems and reduce native biodiversity, growing on roadsides and lakeshores and becoming a problem in relatively dry areas [17,18]. Several studies are necessary to find efficient and sustainable alternatives to synthetic herbicides, whose persistent use has led to the arousal of multiple problems [5], such as the appearance of resistant weeds and toxicity in humans and other living organisms, as well as the persistence of residues in the environment that affect soil, air, the surrounding environment, ground water and crops [19,20,21]. The development of natural herbicides based on essential oils could decrease these negative impacts, mainly by counteracting resistant weeds, since it is difficult to develop resistance using mixtures of natural components with different mechanisms of action. In this sense, agricultural compositions, including oregano essential oil together with others also belonging to the Lamiaceae family such as *Lavandula*, *Mentha*, *Rosmarinus*, and *Salvia* species, have been elaborated as natural pesticides [22]. Similarly, lemongrass essential oil (*Cymbopogon citratus*, Poaceae) has been included as a principal ingredient in a natural herbicide invention to control the germination and growth of weeds [23].

On the other hand, it is interesting to demonstrate the selectivity of these eco-friendly active components against weeds and/or invasive species, thereby confirming their harmlessness over food crops. Previous studies demonstrated that winter savory (*Satureja montana* L.) essential oil is effective in the management of *P. oleracea*, *L. multiflorum*, and *E. crus-galli*, without being pernicious to the food crops maize, rice, and tomato. Similarly, peppermint (*Mentha piperita* L.) essential oil could be used to control *L. multiflorum* in rice (*Oryza sativa* L.) [24]. *E. citriodrora* essential oil affected seed germination in *Amaranthus viridis* L., without harming the food crops commonly affected by the weed *Triticum aestivum* L., *Zea mays* L., and *Raphanus sativus* L. [11]. However, this essential oil also produced a cytotoxic effect against food crops such as *Lactuca sativa* L. [25], and other essential oils, such as wintergreen (*Gaultheria procumbens* L.) essential oil with methyl salicylate (99.6%) as the main compound, could be employed in the control of the invasive species *Cortaderia seollana* (Schult. & Schult. f.) Asch. & Graebn, and *Nicotiana glauca* Graham [26].

The tested essential oils have been selected for their pharmacological or biological properties as well as for their chemical profile. Regarding this, *Eucalyptus citriodora* L. essential oil showed moderate antioxidant action, potent antimicrobial activity against bacteria and yeasts [27,28], and insect-repellent capacity when included in insect-repellent compositions [29]. Recently, citronellal, the main component of *E. citriodora* essential oil, has been encapsulated individually in different types of cyclodextrins to maintain its properties for longer [30]. Lavender (*Lavandula angustifolia* Mill.) essential oil and its main compounds 1,8-cineole and linalool have also shown antimicrobial potential, with synergistic effects with other common antimicrobial agents [31,32,33]. The antibacterial activity of *L. angustifolia* essential oil can be improved by being embedded with cyclodextrin, because this increases its water solubility and reduces its volatility [34]. The antimicrobial activity of *Pinus sylvestris* L. essential oil has been also well-established. In addition, *P. sylvestris* essential oil has shown a higher insect larvicidal potential against *Drosophila melanogaster* Meigen than other *Pinus* species, such as *P. peuce*, *P. nigra* subsp. *nigra* and *P. musco* subsp. *musgo* [35,36]. The high antimicrobial activity may be due to α- and β-pinene, the major compounds in *P. sylvestris* essential oil, which have already shown their antibacterial and antifungal potential. Indeed, both pinenes have been combined with commercial antimicrobials resulting in a reduction of their minimum inhibitory concentration and toxicity [37].

Since the phytotoxic effects differ remarkably with the chemical composition and the chemical composition of an essential oil depending on certain intrinsic and extrinsic factors [38] such as the extraction method [39], geographic location [40,41,42], temperature, and drying period, as well as harvesting time [43,44], the aims of this study were: (i) to determine through Gas Chromatography–Mass Spectrometry analysis the chemical composition of commercial *Eucalyptus citriodora* Hook, *Lavandula angustifolia* Mill., and *Pinus sylvestris* L. essential oils; (ii) to test the in vitro phytotoxic activity of these essential oils against the seed germination and seedling growth of the weeds *P. oleracea*, *L. multiflorum* and *E. crus-galli*, to evaluate their herbicidal activity, as well as on food crops such as tomato (*Solanum lycopersicum* L.) and cucumber (*Cucumis sativus* L.), to know its harmful effects on crops; and (iii) to test the same against the invasive species *Nicotiana glauca* Graham, potential reservoir of important viruses, including cucumber mosaic virus and tomato infectious chlorosis virus, which causes economic losses for commercial tomato production.

## 2. Results and Discussion

### 2.1. Chemical Composition of E. citriodora, L. angustifolia, and P. sylvestris Essential Oils

Twenty-seven (98.6%), 60 (97.6%) and 38 (99.1%) compounds in commercial *E. citriodora*, *L. angustifolia*, and *P. sylvestris* essential oils, respectively, were identified by Gas Chromatography–Mass Spectrometry analysis. Components were clustered (Table 1) in a homologous series of monoterpene hydrocarbons, oxygenated monoterpenes, sesquiterpene hydrocarbons, oxygenated sesquiterpenes, oxygenated diterpenes, aromatic compounds, and others, and listed according to Kovat’s retention index [45] calculated in GC on an apolar HP-5MS column.

Citronellal was the major component in *E. citriodora* essential oil (88.0 ± 0.8%), whereas linalool (38.7 ± 0.1%) together with 1,8-cineole (26.5 ± 0.1%) and camphor (14.2 ± 0.1%) were the main components in *L. angutifolia* essential oil (Table 1). Citronellal was also the main compound in *E. citriodora* essential oils of different origins and light conditions [46,47], making *E. citriodora* distinct from other *Eucalyptus* species, such as *E. camaldulensis* Dehnh [48].

However, qualitative and quantitative differences in the chemical composition of lavender essential oil have been reported depending on the biological raw material, level of dryness, extraction method, and origin. Previous studies showed that the drying process reduced the concentrations of the principal components in *L. angustifolia* essential oil obtained from flowers and aerial parts [49]. Similarly, the extraction method employed varied the content of linalool, detected in a much higher content using hydrodistillation in comparison to supercritical CO_2_ and hexane extraction [50]. Furthermore, *L. angustifolia* essential oil hydrodistilled from aerial parts coming from Yazd (Iran) had a dissimilar chemical composition to our results, with 1,8-cineole, camphor, and borneol as its main components. This was also dissimilar to a sample from lavender essential oil obtained from the inflorescences of *L. angustifolia* “Sevtopolis” cultivated in western Romania, which had linalyl acetate (40.7%), linalool (22.5%), caryophyllene (8.9%), and lavandulyl acetate (7.5%) as its principal components [51]. In addition, it has been recently observed that the chemical composition of *L. angustifolia* essential oil can be modified by the application of gold and silver metals as elicitors, decreasing lower-molecular-weight compounds such as α- and β-pinene, camphene, δ-3-carene, *p*-cymene, 1,8-cineole, pinocarveol, etc., which are replaced by higher-molecular-weight compounds such as α-cadinol 9-cedranone, cadalene, α-bisabolol, and (E,E)-farnesol, varying the biological properties [52]. Other presentations, such as hydrolates, produced a reduction in volatile compound content and a reduction in antioxidant activity [53].

On the other hand, α-pinene (25.6 ± 0.2%), limonene (18.5 ± 0.2%), and β-pinene (15.9 ± 0.1%) were the main components (Table 1) in *P. sylvestris* essential oil. The predominance of monoterpene hydrocarbons is a characteristic feature of essential oils obtained from the Pinaceae family, e.g., monoterpene hydrocarbons were the major fraction of *P. nigra* var. *italica* essential oil (63.4%), with α-pinene as its most abundant compound (49.0%) [54], as well as in the essential oil obtained from the hydrodistillation of *P. armandii*, *P. nigra* and *P. halepensis* cones with α-pinene, limonene, and β-pinene as principal components [55,56].

The remarkable concentration of limonene in *P. sylvestris* essential oil may also contribute to the antimicrobial properties. In fact, a limonene emulsion has been effectively stabilized by *Ulva fasciata* Delile polysaccharide to be applied to food to avoid foodborne pathogen contamination and consequently prolong shelf-life [57,58].

In contrast to our results, sesquiterpene hydrocarbons have been described as one of the most representative phytochemical groups in the *Pinus* genus together with monoterpene hydrocarbons, with germacrene D or β-caryophyllene being the most characteristic compounds within the group [59]. Thus, essential oils obtained from the needles of other *Pinus* species such as *P. roxburghaii* contain large amounts of α-pinene (29.3%) and β-caryophyllene (21.9%), whereas α-pinene (35.4%) and germacrene D (28.1%) were the main components of the *P. nigra* subsp. *nigra* essential oil [36,60].

### 2.2. Seed Germination Inhibition of P. oleracea, L. multiflorum, E. crus-galli, Tomato, Cucumber and N. galuca with E. citriodora, L. angustifolia and P. sylvestris Essential Oils

The in vitro phytotoxic potential of *E. citriodora*, *L. angustifolia*, and *P. sylvestris* essential oils was evaluated against seed germination in weeds (*P. oleracea*, *L. multiflorum*, and *E. crus-galli*), as well as against two Mediterranean food crops (tomato and cucumber), and the invasive species *N. glauca*, at several doses (0.125, 0.25, 0.50, and 1 µL/mL) (Table 2 and Table 3).

Regarding the phytotoxic effects of the selected essential oils against weeds, variability at the intraindividual level was observed in the seed germination percentage, without statistical significance. *E. citriodora* and *P. sylvestris* did not cause a significant inhibition of seed germination in either *P. oleracea*, *L. multiflorum*, or *E. crus-galli* at any assayed dose (0.125, 0.25, 0.5, and 1 µL/mL) (Table 2). However, citronellal, the main compound in *E. citriodora* essential oil analyzed here, showed seed germination inhibition against other weeds including *Ageratum conyzoides* L., *Chenopodium album* L., *Parthenium hysterophorus* L., *Malvastrum coromandelianum* (L.) Garcke, *Cassia occidentalis* L., and *Philaris minor* Retz. at 100 µg/g [61]. In relation to food crops, citronellal was able to inhibit seed germination in *L. sativa*, reaching 49–15% of the control [62], as well as seed germination in tomato at a percentage of 64.8% at the highest tested dose (1 µL/mL). *P. sylvestris* with α-pinene (25.6%) as the main compound showed phytotoxic effects in seed germination in cucumber at all applied doses (0.125, 0.25, 0.50, and 1 µL/mL), while another *Eucalyptus* species (*E. tereticornis*), which contained principally α-pinene (34.5%), produced selective toxicity against the seed germination of *E. crus-galli* without affecting the rice crop to the same extent [63]. 

By contrast, although *L. angustifolia* essential oil did not exhibit a significant inhibition of seed germination in *P. oleracea*, it achieved a remarkable reduction of seed germination in both *L. multiflorum* and *E. crus-galli*. This fact may be because *L. angustifolia* essential oil, among the essential oils analyzed here, contains the largest number (27 vs. 12 and 14) of oxygenated compounds, especially oxygenated monoterpenes (1,8-cineole, linalool, camphor, borneol, α-terpineol) that have shown higher herbicidal properties [64].

*L. multiflorum* showed more susceptibility to the phytotoxic effect of *L. angustifolia* essential oil, which decreased the percentage of seed germination in a dose-dependent manner, reaching increasing percentages of inhibition of 44.6% and 63.1% at the highest applied doses (0.5 and 1 µL/mL, respectively) (Table 2). The fact that *L. multiflorum* showed a certain sensitivity to *L. angustifolia* essential oil could be interesting in the research of essential oils as natural alternatives to synthetic herbicides used against *L. multiflorum*, which have caused the emergence of resistance in this weed [65,66,67]. In other studies, peppermint (*Mentha piperita* L.) essential oil caused a total inhibition of seed germination in *L. multiflorum* at a range of concentrations between 0.125 and 1 µL/mL, and caused inhibition in food crops (maize, rice, and tomato). In our study, *L. angustifolia* essential oil produced less phytotoxic effects in food crops. The seed germination of tomato was reduced at the highest dose tested, at a percentage of 69.02% (vs. 99.97% with peppermint essential oil) with respect to the control [24], while the seed germination of cucumber was not significantly inhibited at any assayed dose (0.125, 0.25, 0.50, and 1 µL/mL).

Seed germination in *E. crus-galli* also showed a certain weakness to exposure to *L. angustifolia* essential oil at the highest tested dose (1 µL/mL), with a percentage of inhibition of 18.3% (Table 2).

Tomato was more sensitive to *E. citriodora* and *L. angustifolia* essential oils with similar remarkable reduction at the highest applied dose (1 µL/mL), reaching 64.8 and 69.0% reduction, respectively (Table 2).

In general, cucumber was more resistant than tomato to the phytotoxic effects of the three commercial essential oils applied, without inhibitory effect at any assayed dose (0.125, 0.25, 0.5, and 1 µL/mL) with *E. citriodora* and *L. angustifolia* essential oils, and only a low percentage of inhibition (10.00%) at the highest tested dose (1 µL/mL) with *P. sylvestris* essential oil (Table 2).

In addition, the two essential oils richest in oxygenated monoterpenes, *E. citriodora* and *L. angustifolia* (94.7% and 85.5%, respectively), showed similar significant phytotoxic effects against seed germination in the invasive species *N. glauca*, but with a lower percentage in relation to weeds (27.5% and 29.7%) at the highest tested dose (1 µL/mL) (Table 3). Therefore, the various main compounds of an essential oil can produce similar phytotoxic effects against different species.

### 2.3. Seedling Growth Inhibition of P. oleracea, L. multiflorum, E. crus-galli, Tomato, Cucumber and N. glauca with E. citriodora, L. angustifolia, and P. sylvestris Essential Oils

**Figure 1:**
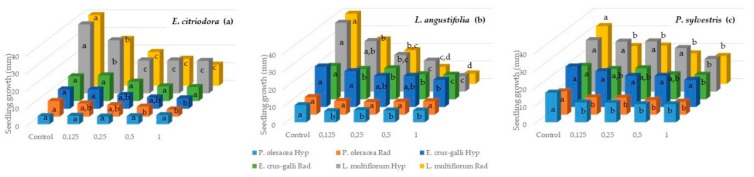
Phytotoxic effect of *E. citriodora* (**a**), *L. angustifolia* (**b**), and *P. sylvestris* (**c**) essential oils on the seedling growth (hypocotyl and radicle) of *P. oleracea*, *L. multiflorum*, and *E. crus-galli*. Values are mean percentages of five replications, after 14 days of incubation. Doses 0.125–1 µL/mL. Different letters indicate significant difference at *p* < 0.05, according to T3 Dunnett and Tukey tests.

The hypocotyl growth of *P. oleracea* was not significantly reduced by *E. citriodora* essential oil at any applied dose (0.125, 0.25, 0.5, and 1 µL/mL); however, this essential oil was able to reduce radicle development at the highest assayed doses (0.5 and 1 µL/mL), reaching 36.4% and 43.2% reduction compared to the control, respectively (Figure 1a). The root growth of *P. oleracea* was more sensitive than shoot growth to citronellal, according to previous studies, due to the mitotic activity of growing root tip cells [61]. However, other mechanisms would have been present with other essential oils because the roots were not significantly affected at doses that produced toxic effects in the hypocotyl. Therefore, the hypocotyl elongation of *P. oleracea* was remarkably reduced from the lowest applied dose (0.125 µL/mL) of *L. angustifolia* (Figure 1b) and *P. sylvestris* (Figure 1c) essential oils with respect to control, reaching a decrease of 30.6% and 39.3%, respectively, at the highest tested dose (1 µL/mL), whereas radicle development was not significantly affected by *L. angustifolia* essential oil (Figure 1b), yet *P. sylvestris* essential oil achieved a significant reduction in radicle development (26.0–44.4%) from the lowest to the highest applied dose (0.125–1 µL/mL) (Figure 1c).

Regarding the seedling evolution of *E. crus-galli* after the application of the essential oils, it was observed that *L. angustifolia* essential oil was the most harmful for *E. crus-galli* seedling growth, as it decreased its hypocotyl in a high percentage (76.7%) at the highest assayed dose (1 µL/mL), as well as the radicle, in a dose-dependent manner, also reaching a considerable percentage (69.9%) at the highest applied dose (1 µL/mL) (Figure 1b). Although *E. citriodora* essential oil did not influence radicle elongation at any applied dose (0.125, 0.25, 0.5, and 1 µL/mL), hypocotyl growth was significantly affected at 1 µL/mL, reaching a reduction percentage of 46.1% in comparison to control (Figure 1a). *P. sylvestris* essential oil was the least phytotoxic essential oil, with no reduction in the hypocotyl development of *E. crus-galli* at any dose (0.125, 0.25, 0.5, and 1 µL/mL), and a low percentage of radicle elongation reduction (26.5%) at the highest dose (Figure 1c). However, other studies demonstrated that α-pinene exhibited a certain inhibition of the early root growth of other weeds such as *Cassia occidentalis* (L.) Link., as well as oxidative damage in root tissue [10]. Similarly, the compound β-pinene was shown to be responsible for the disruption of membrane integrity, the enhancement of peroxidation and electrolyte leakage in *Phalaris minor* and particularly in *E. crus-galli* [68].

Both the hypocotyl and radicle development of *L. multiflorum* were significantly inhibited by *E. citriodora* (Figure 1a) and *L. angustifolia* (Figure 1b), which caused a strong dose-dependent reduction, reaching 52.3–53.0% and 60.6–75.4% at 0.25-1 µL/mL, and 55.1–77.5 and 80.1–87.8% at 0.5-1 µL/mL, respectively (Figure 1a,b). *P. sylvestris* essential oil did not significantly affect hypocotyl growth, but it did inhibit the radicle development of *L. multiflorum* in the range of 0.125 to 1 µL/mL without distinction between doses, reaching 51.67% reduction at the highest dose assayed (Figure 1c). With *L. multiflorum*, it was corroborated that α-pinene, the main compound of *P. sylvestris* essential oil analyzed here, affects root development to a greater extent than hypocotyl, as it was also able to inhibit the radicle growth of other weed species such as *Amaranthus viridis* L., *Triticum aestivum* L., *Pisum sativum* L., *Cicer arietinum* L., and especially *C. occidental*, which demonstrated solute leakage, lipid peroxidation and the generation of reactive oxygen species upon α-pinene exposure [10].

**Figure 2:**
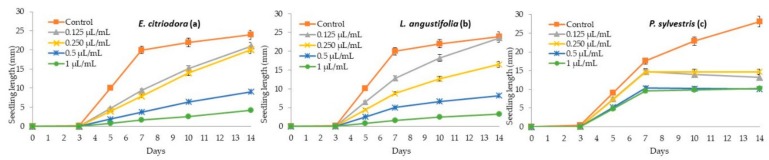
Phytotoxic effect of *E. citriodora* (**a**), *L. angustifolia* (**b**), and *P. sylvestris* (**c**) essential oils at 0.125, 0.25, 0.5, and 1 µL/mL on the seedling growth (hypocotyl + radicle) of tomato.

**Figure 3:**
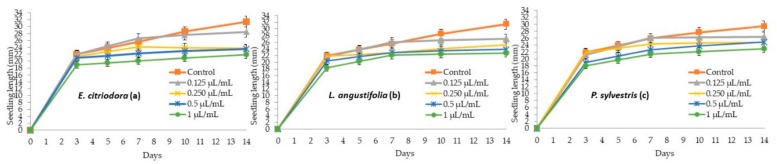
Phytotoxic effect of *E. citriodora* (**a**), *L. angustifolia* (**b**), and *P. sylvestris* (**c**) essential oils at 0.125, 0.25, 0.5, and 1 µL/mL on the seedling growth (hypocotyl + radicle) of cucumber.

Regarding the sensitivity of the seedling growth of food crops to essential oils, it was observed that tomato was more susceptible than cucumber to *E. citriodora*, *L. angustifolia*, and *P. sylvestris* essential oils (Table 4, Figure 2 and Figure 3). Both the hypocotyl and radicle development of tomato were significantly reduced in a dose-dependent manner, reaching elevated reduction percentages at the highest applied dose (1 µL/mL) of *E. citriodora* (89.7 and 79.4%) and *L. angustifolia* (93.2% and 83.4%) essential oils (Figure 2a,b). *L. sativa* was another food crop that showed high sensitivity to the application of citronellal [62], and *E. citriodora* essential oil affected meristematic cells, decreasing the germination and seedling growth of this food crop [25].

Again, *P. sylvestris* was the least phytotoxic essential oil, but also showed a significant inhibition of hypocotyl and radicle development, measuring 72.2% and 62.9%, respectively, at the dose of 1 µL/mL (Table 4).

On the other hand, none of the assayed essential oils significantly affected the hypocotyl growth of cucumber (Table 4). However, the radicle development of cucumber was significantly reduced, up to a percentage of 42.4%, 37.8%, and 28.0% at the highest applied doses of *E. citriodora*, *L. angustifolia*, and *P. sylvestris* essential oils (Table 4).

Finally, *E. citriodora* essential oil showed more phytotoxic effects than *L. angustifolia* essential oil in both the hypocotyl and radicle elongation of the invasive species *N. glauca*, reaching percentage reductions of 85.8% and 69.4% *versu*s 51.8% and 37.6%, respectively (Table 3).

## 3. Materials and Methods 

### 3.1. Essential Oils

Commercial samples of *Eucalyptus citriodora* Hook (Batch: OF25830; Exp. Date: 02/2022), *Lavandula angustifolia* Mill. (Batch: 0082842; Exp. Date: 30/11/2020), and *Pinus sylvestris* L. (Batch: 0065144; Exp. Date: 08/08/2018) essential oils obtained from the hydrodistillation of leaves, flowers, and needles, respectively, were supplied by Pranarôm S.A. (*E. citriodora*) and Guinama (Valencia, Spain). The essential oils were stored at 4 °C until chemical analysis and phytotoxic assays were carried out.

### 3.2. Weeds, Food Crops, and Invasive Species Seeds

Mature seeds of the weeds common purslane (*Portulaca oleracea* L.), Italian ryegrass (*Lolium multiflorum* Lam.), and barnyardgrass (*Echinochloa crus-galli* (L.) Beauv.) were purchased from Herbiseed (website: www.herbiseed.com).

Mature seeds of the food crops “Muchamiel” tomato (*Solanum lycopersicum* L.) and cucumber (*Cucumis sativus* L.) were obtained from Intersemillas S.A.

Mature seeds of the invasive species tree tobacco (*Nicotiana glauca* Graham) were supplied by the Botanical Garden of Valencia.

### 3.3. Gas Chromatography–Mass Spectrometry Analysis

Gas Chromatography–Mass Spectrometry analysis was carried out using a 5977A Agilent mass spectrometer and a gas chromatograph (Agilent 7890B, Valencia, España) apparatus equipped with an Agilent HP-5MS (30 m long and 0.25 mm i.d. with 0.25 µm film thickness) capillary column (95% dimethylpolysiloxane/5% diphenyl). The column temperature program was 60 °C for a duration of 5 min, with 3 °C/min increases up to 180 °C, then 20 °C/min increases up to 280 °C, which was maintained for 10 min. The carrier gas was helium at a flow rate of 1 mL/min. Split-mode injection (ratio 1:30) was employed. Mass spectra were collected over the *m/z* range 30–650 with an ionizing voltage of 70 eV. The resulting individual compounds were identified by MS and their identity was confirmed by comparison of their Kovat’s retention index, calculated using co-chromatographed standard hydrocarbons relative to C_8_–C_32_ *n*-alkanes and mass spectra with reference samples or with data already available in the NIST 11 mass spectral library and in the literature [45].

### 3.4. In Vitro Assays: P. oleracea, L. multiflorum, E. crus-galli, Tomato, Cucumber, and N. glauca Seed Germination and Seedling Growth with Essential Oils

Sets of 20 seeds each with five replicates per treatment were homogenously distributed in Petri dishes (9 cm diameter) between two layers of filter paper (Whatman No.1). The lower filter papers were moistened with 4 mL of distilled water and the upper ones with 0 (control), 0.125, 0.250, 0.5, and 1 µL/mL of *E. citriodora*, *L. angustifolia*, and *P. sylvestris* essential oils, homogeneously distributed in the filter paper with a micropipette (Merck®, Valencia, España). Therefore, the seeds were in contact directly with moistened filter papers and indirectly with the vapors of the essential oils. Petri dishes were sealed with parafilm and incubated in an Equitec EGCS 301 3SHR model germination chamber, according to previous assays [69], alternating between 30.0 ± 0.1 °C 16 h of light and 20.0 ± 0.1 °C 8 h of darkness, with and without humidity. To evaluate the herbicidal activity of the essential oils, the number of germinated seeds was counted and compared with that of untreated seedlings. The emergence of the radicle (≥1 mm) was used as an index of germination, and seedling length (hypocotyl and/or radicle) data were recorded after 3, 5, 7, 10, and 14 days in each replicate.

### 3.5. Statistics

Experiments were performed in vitro with five replicates. Data were subjected to one-way analysis of variance (ANOVA) using SPSS statistics 24 software. Tukey’s *post hoc* test was used when variances remained homogeneous (Levene’s test) and T3 Dunnett’s *post hoc* test was employed if not, assuming equal variances. Differences were considered to be significant at *p* ≤ 0.05.

## 4. Conclusions

In this study, the potential of *E. citriodora*, *L. angustifolia*, and *P. sylvestris* essential oils as eco-friendly alternatives to synthetic herbicides was investigated. *L. angustifolia* essential oil, with a high content of the oxygenated monoterpenes linalool (38.7 ± 0.1%), 1,8-cineole (26.5 ± 0.1%), and camphor (14.2 ± 0.1%), affected seed germination and development of *L. multiflorum*, *E. crus-galli*, and *N. glauca* without any significant phytotoxic effect on cucumber seed germination. *E. citriodora*, with a high content of the oxygenated monoterpene citronellal (88.0 ± 0.8%), showed more phytotoxic effects than *L. angustifolia* on the control of *N. glauca*. Lavender essential oil represents an effective pre-emergent treatment for *L. multiflorum* affecting cucumber crops, and *E.citriodora* essential oil could be used in both pre- and post-management of the invasive species *N. glauca*.

## Figures and Tables

**Table 1:** Chemical compositions of commercial *E. citriodora*, *L. angustifolia*, and *P. sylvestris* essential oils.

RI_Cal_	RI_Ref_	Compound	*E. citriodora* Relative Area (%)	*L. angustifolia* Relative Area (%)	*P. sylvestris* Relative Area (%)
**Monoterpene hydrocarbons**			1.5 ± 0.1	7.8 ± 0.1	74.4 ± 0.3
924	926	Tricyclene	-	t	0.1 ± 0.0
926	930	α-Thujene	t	-	-
939	939	α-Pinene	0.2 ± 0.0	2.5 ± 0.0	25.6 ± 0.2
953	954	Camphene	-	0.7 ± 0.0	6.4 ± 0.1
977	975	Sabinene	t	0.3 ± 0.0	-
985	979	β-Pinene	0.5 ± 0.0	2.4 ± 0.0	15.9 ± 0.1
980	987	3-*p*-Menthene	-	-	0.2 ± 0.0
998	990	Myrcene	0.1 ± 0.0	0.5 ± 0.0	3.5 ± 0.0
1012	1011	δ-3-Carene	-	-	0.6 ± 0.0
1020	1017	α-Terpinene	t	0.1 ± 0.0	2.3 ± 0.0
1021	1024	*p*-Cymene	t	0.5 ± 0.0	0.9 ± 0.0
1028	1029	Limonene	t	-	18.5 ± 0.2
1043	1037	*cis*-Ocimene	-	0.1 ± 0.1	-
1053	1050	*trans*-β-Ocimene	0.1 ± 0.0	0.1 ± 0.0	-
1056	1059	γ-Terpinene	0.2 ± 0.0	0.3 ± 0.0	0.1 ± 0.0
1090	1088	Terpinolene	0.3 ± 0.0	0.3 ± 0.0	0.3 ± 0.0
Oxygenated monoterpenes			94.7 ± 1.2	85.5 ± 0.1	23.4 ± 0.3
1029	1031	1,8-Cineole	0.3 ± 0.0	26.5 ± 0.0	2.1 ± 0.2
1051	1056	Bergamal	0.1 ± 0.0	-	-
1070	1070	*cis*-Sabinene Hydrate	-	0.2 ± 0.0	-
1076	1072	*cis*-Linalool Oxide	-	0.1 ± 0.0	-
1095	1096	Linalool	0.1 ± 0.0	38.7 ± 0.1	t
1098	1099	α-Pinene Oxide	-	-	0.1 ± 0.0
1104	1108	*cis*-Rose Oxide	0.1 ± 0.0	-	-
1122	1125	*trans*-Rose Oxide	t	-	-
1129		Plinol C	-	0.4 ± 0.1	-
1144	1146	Camphor	-	14.2 ± 0.1	0.5 ± 0.0
1150	1149	Isopulegol	4.3 ± 1.1	-	-
1154	1153	Citronellal	88.0 ± 0.8	-	-
1158	1159	*iso*-Isopulegol	0.5 ± 0.1	-	-
1159	1160	Isoborneol	-	0.4 ± 0.0	-
1168	1166	δ-Terpineol	-	0.3 ± 0.0	-
1170	1169	Borneol	-	1.3 ± 0.0	-
1179	1177	Terpinen-4-ol	-	0.3 ± 0.0	t
1184	1182	*p*-Cymen-8-ol	-	0.1 ± 0.0	-
1187	1185	Cryptone	-	t	-
1188	1188	α-Terpineol	-	1.6 ± 0.0	0.1 ± 0.0
1196	1195	Myrtenal	-	0.1 ± 0.0	-
1197	1199	γ-Terpineol	-	0.2 ± 0.0	-
1212	1220	α-Fenchyl Acetate	-	-	0.1 ± 0.0
1231	1229	Nerol	-	0.1 ± 0.0	-
1256	1252	Piperitone	-	t	-
1258	1252	Geraniol	-	0.2 ± 0.0	-
1260	1257	Linalool Acetate	-	0.5 ± 0.0	t
1287	1288	Bornyl Acetate	-	0.1 ± 0.0	17.9 ± 0.0
1311	1313	Citronellic Acid	0.1 ± 0.0	-	-
1325		β-Terpinyl Acetate	-	-	0.1 ± 0.0
1345	1349	α-Terpinyl Acetate	-	-	2.6 ± 0.0
1348	1352	Citronellyl Acetate	1.3 ± 0.1	-	-
1368	1361	Neryl Acetate	-	0.2 ± 0.0	-
1468	1468	Linalool Isovalerate	-	0.1 ± 0.0	-
1512	1511	Lavandulyl 2-Methyl Butanoate	-	0.1 ± 0.0	-
Sesquiterpene hydrocarbons			2.1 ± 0.2	3.3 ± 0.0	0.7 ± 0.0
1330	1338	δ-Elemene	-	-	t
1377	1376	α-Copaene	-	t	-
1381	1381	Daucene	-	t	-
1383	1388	β-Bourbonene	-	0.1 ± 0.0	-
1385	1390	β-Elemene	-	-	t
1391	1391	7-*epi*-Sesquithujene	-	0.1 ± 0.0	-
1403	1405	Sesquithujene	-	0.1 ± 0.0	-
1407	1407	Longifolene	-	-	0.1 ± 0.0
1409	1409	α-Gurjunene	-	0.1 ± 0.0	-
1410	1411	α-Cedrene	-	-	0.1 ± 0.0
1420	1419	β-Caryophyllene	2.0 ± 0.2	1.8 ± 0.0	0.4 ± 0.0
1427	1434	α-*trans*-Bergamotene	-	0.1 ± 0.0	-
1435	1436	γ-Elemene	-	-	t
1454	1454	α-Humulene	-	0.1 ± 0.0	t
1460	1456	*trans*-β-Farnesene	-	0.2 ± 0.0	-
1470	1472	Dauca-5,8-diene	-	t	-
1481	1479	γ-Muurolene	-	0.3 ± 0.0	-
1495	1500	Bicyclogermacrene	0.1 ± 0.0	-	-
1500	1500	α-Muurolene	-	-	t
1510	1505	β-Bisabolene	-	0.2 ± 0.0	-
1514	1513	γ-Cadinene	-	0.2 ± 0.0	t
1524	1522	*trans*-Calamenene	-	t	-
1525	1523	δ-Cadinene	-	t	0.1 ± 0.0
		Germacrene B	-	-	t
Oxygenated sesquiterpenes			t	0.3 ± 0.0	0.3 ± 0.0
1582	1583	Caryophyllene Oxide	t	0.2 ± 0.0	t
1599	1600	Cedrol	-	-	0.1 ± 0.0
1641	1640	*epi*-α-Cadinol	-	0.1 ± 0.0	-
1684	1685	α-Bisabolol	-	t	-
Oxygenated Diterpenes			-	-	0.1 ± 0.0
1985	1987	Manool Oxide	-	-	0.1 ± 0.0
Aromatic compounds			0.1 ± 0.0	t	0.3 ± 0.0
1247	1250	*p*-Anis Aldehyde	-	-	0.3 ± 0.0
1351	1359	Eugenol	0.1 ± 0.0	-	-
1434	1434	Coumarin	-	t	-
Others			0.1 ± 0.0	0.5 ± 0.1	-
868	870	*n*-Hexanol	-	t	-
910		Isobutyl Isobutyrate	0.1 ± 0.0	-	-
983	979	1-Octen-3-ol	-	t	-
1008		Isoamyl Isobutyrate	t	-	-
1194	1192	Hexyl Butanoate	-	0.1 ± 0.0	-
1234	1332	Hexyl Tiglate	-	0.1 ± 0.0	-
1244	1244	Hexyl Isovalerate	-	0.3 ± 0.0	-
Total			98.6 ± 1.2	97.6 ± 0.2	99.1 ± 0.0

RI_Cal_: retention index relative to C_8_-C_32_ *n*-alkane on HP-5MS column; RI_Ref_: retention index reported in Adams 2007 [45]; t: trace amounts < 0.05. Values are means ± standard deviation of the three samples.

**Table 2:** In vitro phytotoxic effect of different doses of *E. citriodora*, *L. angustifolia*, and *P. sylvestris* essential oils on *Portulaca oleracea*, *Lolium multiflorum*, *Echinochloa crus-galli,* tomato, and cucumber seed germination.

Seed Germination (% ± S.E.)					
* Dose	***E. citriodora* essential oil**				
	***P. oleracea***	***L. multiflorum***	***E. crus-galli***	*Tomato*	*Cucumber*
Control	74.0 ± 4.6 a	65.0 ± 6.9 a	69.0 ± 2.9 a	71.0 ± 2.5 a	99.0 ± 1.0 a
0.125	80.0 ± 2.2 a	67.0 ± 4.4 a	74.0 ± 3.7 a	71.0 ± 4.3 a	98.0 ± 1.2 a
0.25	76.0 ± 2.9 a	52.0 ± 2.0 a	72.0 ± 2.6 a	73.0 ± 3.4 a	95.0 ± 2.2 a
0.5	74.0 ± 4.3 a	58.0 ± 2.6 a	61.0 ± 4.6 a	61.0 ± 3.7 a	97.0 ± 1.2 a
1	81.0 ± 6.2 a	57.0 ± 7.2 a	72.0 ± 3.7 a	25.0 ± 11.3 b	96.0 ± 1.8 a
Dose	***L. angustifolia* essential oil**				
Control	74.0 ± 3.7 a	65.0 ± 6.9 a	71.0 ± 4.3 a	71.0 ± 2.5 a	99.0 ± 1.0 a
0.125	69.0 ± 5.3 a	65.0 ± 3.2 a	71.0 ± 2.8 a	73.0 ± 4.4 a	97.0 ± 1.2 a
0.25	67.0 ± 2.0 a	50.0 ± 2.7 a,b	72.0 ± 2.6 a	58.0 ± 4.1 a,b	98.0 ± 2.0 a
0.5	66.0 ± 5.8 a	36.0 ± 8.4 b,c	72.0 ± 3.4 a	41.0 ± 13.2 b,c	97.0 ± 1.2 a
1	69.0 ± 3.7 a	24.0 ± 7.0 c	58.0 ± 2.6 b	22.005.8 c	94.0 ± 1.9 a
Dose	***P. sylvestris* essential oil**				
Control	75.0 ± 7.1 a	67.0 ± 2.0 a	74.0 ± 3.3 a	68.0 ± 3.4 a	100.0 ± 0.0 a
0.125	74.0 ± 3.7 a	65.0 ± 8.8 a	69.0 ± 7.0 a	67.0 ± 4.4 a	94.0 ± 2.9 a,b
0.25	71.0 ± 2.9 a	65.0 ± 5.0 a	74.0 ± 1.9 a	67.0 ± 4.1 a	94.0 ± 1.9 a,b
0.5	71.0 ± 1.9 a	58.0 ± 5.2 a	74.0 ± 4.6 a	66.0 ± 3.7 a	95.0 ± 1.6 a,b
1	68.0 ± 2.6 a	51.0 ± 12.8 a	75.0 ± 5.0 a	64.0 ± 3.7 a	90.0 ± 2.3 b

Values are the mean percentage of five replications ± standard error, after 14 days of incubation. Means followed by different letters in the same column indicate significant difference at *p* < 0.05, according to T3 Dunnett and Tukey tests. * Dose: µL/mL.

**Table 3:** In vitro phytotoxic effect of different doses of *E. citriodora* and *L. angustifolia* essential oils on the seed germination and seedling growth of *N. glauca.*

**Concentration (µL/mL)**	***E. citriodora***		
	**Germination**	**Hypocotyl**	**Radicle**
Control	91.0 ± 3.3 a	2.5 ± 0.2 a	3.1 ± 0.3 a
0.125	72.00 ± 6.8 a	1.4 ± 0.3 b	2.5 ± 0.4 a
0.25	68.0 ± 9.0 a	1.4 ± 0.3 b	2.5 ± 0.4 a
0.5	67.003.4 a	1.3 ± 0.2 b	2.5 ± 0.3 a
1	66.0 ± 4.7 b	0.4 ± 0.1 c	1.0 ± 0.3 b
**Concentration (µL/mL)**	***L. angustifolia***		
	**Germination**	**Hypocotyl**	**Radicle**
Control	91.0 ± 3.3 a	2.5 ± 0.3 a	3.1 ± 0.3 a
0.125	81.0 ± 4.0 a	2.6 ± 0.4 a	2.8 ± 0.3 a,b
0.25	81.0 ± 2.9 a	2.6 ± 0.2 a	2.9 ± 0.3 a,b
0.5	78.0 ± 3.7 a	1.8 ± 0.1 a,b	2.4 ± 0.2 a,b
1	64.0 ± 3.7 b	1.2 ± 0.2 b	2.0 ± 0.1 b

Values are the mean of five replications ± error deviation, after 14 days of incubation. Means followed by different letters in the same column indicate significantly difference at *p* < 0.05, according to T3 Dunnett and Tukey tests.

**Table 4:** In vitro phytotoxic effect of different doses of *E. citriodora* (EC), *L. angustifolia* (LA), and *P. sylvestris* (PS) essential oils on tomato (TO) and cucumber (CU) seedling growth.

	* Dose		Control	0.125	0.25	0.5	1
**EC**	**TO**	Hyp	7.3 ± 1.4 a	6.8 ± 1.8 a	5.0 ± 1.2 a,b	2.7 ± 0.5 a,b	0.8 ± 0.4 b
		Rad	16.7 ± 1.5 a	14.1 ± 1.7 a	15.1 ± 1.5 a	6.3 ± 1.3 b	3.4 ± 1.7 b
	**CU**	Hyp	8.4 ± 0.1 a	8.3 ± 0.4 a	8.4 ± 0.2 a	8.4 ± 0.1 a	8.5 ± 0.9 a
		Rad	23.1 ± 1.5 a	20.2 ± 0.5 a	15.3 ± 0.5 b	15.1 ± 0.9 b	13.3 ± 0.4 b
**LA**	**TO**	Hyp	7.3 ± 1.4 a	7.2 ± 0.7 a	4.9 ± 0.9 a,b	2.1 ± 0.8 b,c	0.5 ± 0.3 c
		Rad	16.7 ± 1.5 a	16.4 ± 0.9 a	11.6 ± 1.1 a,b	7.3 ± 2.1 b,c	2.8 ± 2.0 c
	**CU**	Hyp	8.4 ± 0.1 a	8.3 ± 1.0 a	8.1 ± 0.9 a	8.3 ± 0.9 a	8.3 ± 0.04 a
		Rad	23.1 ± 1.5 a	18.9 ± 0.5 b	17.1 ± 0.5 b,c	15.6 ± 1.0 b,c	14.4 ± 0.7 c
**PS**	**TO**	Hyp	12.6 ± 1.6 a	3.8 ± 1.2 b	4.0 ± 0.7 b	3.2 ± 0.7 b	3.5 ± 0.3 b
		Rad	18.1 ± 1.0 a	9.4 ± 1.0 b	10.6 ± 0.5 b	6.8 ± 1.7 b	6.7 ± 0.4 b
	**CU**	Hyp	8.5 ± 0.9 a	8.6 ± 0.2 a	8.4 ± 0.3 a	8.4 ± 0.8 a	7.7 ± 0.9 a
		Rad	21.2 ± 1.0 a	17.8 ± 0.6 a,b	16.3 ± 1.1 b	16.5 ± 0.8 b	15.2 ± 0.8 b

Values are the mean percentage of five replications ± standard error, after 14 days of incubation. Means followed by different letters in the same row indicate significantly difference at *p* < 0.05, according to T3 Dunnett and Tukey tests. * Dose: µL/mL; Hyp: Hypocotyl (mm); Rad: Radicle (mm).
